# Microbiomes, Community Ecology, and the Comparative Method

**DOI:** 10.1128/mSystems.00112-19

**Published:** 2019-05-07

**Authors:** Sarah M. Hird

**Affiliations:** a Department of Molecular and Cell Biology, University of Connecticut, Storrs, Connecticut, USA; b The Institute for Systems Genomics, University of Connecticut, Storrs, Connecticut, USA

**Keywords:** community ecology, comparative methods, evolution, microbiome

## Abstract

Microbiomes contain many levels of biological information, and integrating across the levels creates a holistic understanding of host-microbiome interactions. In my research on the evolution and ecology of avian microbiomes, I use two complementary frameworks: the microbiome as a community and the microbiome as a trait of the host. We draw on classic ecological and evolutionary theory and modern statistical models to advance our understanding in each of these frameworks and then integrate what we have learned into a better understanding of host-associated microbiomes, host evolution, and microbial biodiversity. Ecological theories that bear on processes such as community assembly and metacommunities are well suited for application to microbiomes. Phylogenetic comparative methods can quantify the fit of evolutionary models and detect correlations between traits and correlations between traits and the rate of evolution; these methods allow the inference of evolutionary process from contemporary patterns.

## Abstract (precis)

Microbiomes contain many levels of biological information, and integrating across the levels creates a holistic understanding of host-microbiome interactions. In my research on the evolution and ecology of avian microbiomes, I use two complementary frameworks: the microbiome as a community and the microbiome as a trait of the host.

## PERSPECTIVE

It is clear that the microbiome can influence host biology in myriad ways sufficient to affect host fitness. On the level of the individual host, the microbiome can tip the balance between health and disease, promote or deter proper development, and influence behavior. It is also clear that there is variation within populations in microbiome composition and function, i.e., an axis of individual variation that is exposed to natural selection. If the microbiome can influence the fitness of a host, then we can also ask how it has contributed to the evolutionary trajectory of populations, to the speciation or extinction of lineages, or to the diversification of host biology. These big questions cannot be answered experimentally and attest to the importance of wild organisms and descriptive science for understanding the role of the microbiome in evolutionary biology.

My research is primarily interested in the ecology and evolution of the avian microbiome. Birds are a globally distributed class of vertebrates that encompass immense ecological and morphological diversity. This makes them an excellent comparative system in which to try to discover fundamental rules that shape interactions between hosts and their microbiomes. Their suite of adaptations, particularly feathers and powered flight, have impacted many aspects of avian biology, and the microbiome may be no exception. How have microbes and microbiomes influenced bird biology, including ecology and phylogeny? How have birds influenced the microbiome and its members? To answer these questions and fully understand the processes shaping host-associated microbiomes, we need an ecological understanding of the microbiome as a community of interacting individuals (the microbe’s perspective) and an evolutionary understanding of the microbiome as a trait of the host (the host’s perspective).

## MICROBIOMES AS COMMUNITIES

**FIG 1:**
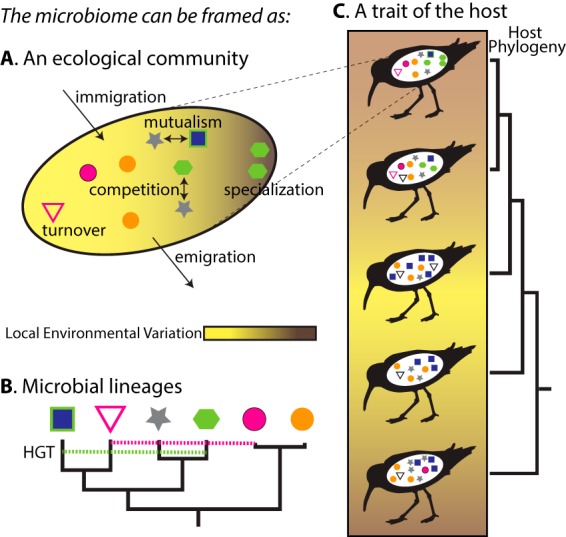
My research integrates several different frameworks with which we can analyze the microbiome in order to understand the role that microbiomes have played in avian diversification. (A) Specifically, the microbiome is an ecological community, consisting of individuals (different shapes) capable of different functions (colors) that are interacting in various ways (e.g., competition) and subject to ecological processes (e.g., migration, ecological drift) and environment. The factors influencing how these communities assemble represent an important consideration, as is how we describe the diversity within the communities, as taxa and functional capabilities are distinct in many communities. (B) Members of the community have unique genomes that are related to one another by phylogeny and nonvertical inheritance of genes (horizontal gene transfer [HGT]). (C) The microbiome is also a trait of a host, with variation across a host species that may relate to environmental variation, the host phylogeny, or the workings of the microbiome as a community (A) or as bacterial lineages (B).

All biodiversity organizes into and functions as members of communities (Fig. 1). How community assembly and persistence are accomplished are fundamental issues in ecology that apply to macroorganisms and microorganisms alike. “Niche-based” community assembly states that organisms have ecological niches that dictate and assure their role in a community. An alternative view is that communities assemble in a neutral way or in a way that is agnostic to ecological niche. The debate concerning the theories of niche-based assembly and neutral assembly of communities is ongoing in the field of ecology, and I am interested in whether and how these theories are supported in avian microbiomes, particularly across body sites with differing properties and purposes. The scale of interrogation can have an impact on whether we discern neutral or niche-based patterns (1), and, given the many hierarchical levels in host-associated microbiomes, the spatial and phylogenetic scales that we consider could matter greatly. Another consideration is intraspecific variability in microbiomes, which exists within the composition and function of microbiomes. Intraspecific variation, compared to interspecific variation, at a given scale may influence our inference of the processes structuring communities and should be quantified when possible (2).

Perhaps microbiomes support both niche-based and neutral components for their community assembly (see, e.g., reference 3). The “competitive lottery model” (4) states that niches in a community can be filled only by organisms with particular functional traits and that any organism that has the trait and arrives first fills that niche. Functional redundancy—where exact species are interchangeable if they perform specific functions—can be a powerful and common force in microbial communities (reviewed in reference 5). Our application of community assembly theory may be greatly improved by considering such biological properties of microbes and microbiomes (reviewed in reference 6). If microbiome assembly is based on function rather than taxonomy, a method that describes taxonomy but not functional capability will not reflect community assembly processes and may positively mislead interpretations.

**In my laboratory.** We are gathering shotgun metagenomic and metatranscriptomic data to better understand the assembly and stability of microbial function in avian microbiomes. We are also pursuing how diversity in these methods compares to the taxonomic diversity estimated using 16S rRNA surveys. To get a complete picture of the microbiome, we are in the nascent stages of incorporating the virome and eukaryotic microorganisms into our diversity estimates as well.

**In the near future.** A better understanding of microbial communities will come with improved methods for describing their fundamental biological units. We are hindered by a lack of an accepted microbial species concept, philosophically and practically. Unfortunately, it seems as if the more (sequence) data we have, the more elusive a definition for species becomes. The use of operational taxonomic units (OTUs) to delimit “species” has been invaluable, but the use of any single marker has limitations. I believe that 16S rRNA surveys will continue to provide important data contributing to our understanding of microbiomes in novel systems. How we complement them to properly describe the functional (and taxonomic) diversity of communities using additional molecular methods is an exciting prospect that is or will soon be within reach for many laboratories as the cost of high-throughput sequencing continues to drop (per base). Databases of full-length 16S rRNA genes, of full-length genomes, and of annotated gene families will continue to grow as novel environments are investigated. Describing novel microbiomes is also important from a basic biodiversity perspective. Discovering the undiscovered requires investment in undescribed hosts or novel ecologies and may have far-reaching benefits, such as adding branches to the tree of life or discovering human life-saving drugs.

## MICROBIOMES AS A TRAIT OF THE HOST

The microbiome can be viewed as a trait of a host (7) (Fig. 1). Understanding the forces shaping trait distributions across species is a fundamental goal of organismal biology. Large-scale patterns in morphological, behavioral, ecological, or genomic traits can indicate the processes acting above the level of the individual and cannot be addressed in laboratory settings or by analyzing a single species. Importantly, many traits are not independent of phylogeny (8), and phylogenetic comparative methods provide one way to account for phylogeny in the study of the distribution of traits among species. Thus, we can use these methods to infer what evolutionary processes have generated the microbiome and to better understand the role that microbiomes have played in host evolution.

One specific use of phylogenetic comparative methods is to assess how well an evolutionary model fits the contemporary distribution of trait values. Given empirical trait values for a set of species and a phylogeny that unites them, we can first infer the state of the trait at all internal nodes of the tree, including the root. Brownian motion models are commonly used as the neutral expectation for how a trait will vary over a particular phylogeny. Information criteria then compare the neutral model against models that incorporate selection or models with no phylogenetic signal at all. In this application, we may discern whether selection need be invoked to explain a trait. A second use of phylogenetic comparative methods is to estimate the correlation between traits or how a trait relates to the environment. Here we may determine factors such as whether a particular host genetic variant is correlated to the microbiome. A third application is to evaluate a trait’s role in speciation and extinction rates. Many options now exist for such tests, including the popular binary-state speciation and extinction (BiSSE) model (9). Here we may ask whether the microbiome contributed to the rapid diversification (or relative stasis) of avian lineages. Interdisciplinary microbiome researchers should be aware of the extensive literature on the strengths and limitations of phylogenetic comparative methods (see, e.g., references 10, 11, and 12) and the statistical assumptions of specific evolutionary models (see, e.g., reference 13).

**FIG 2:**
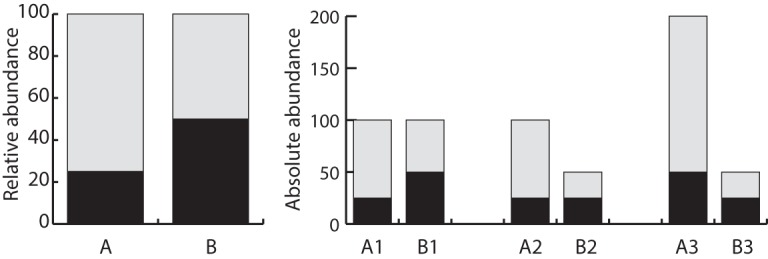
Relative abundance data are compositional, and without also estimating absolute abundance, the relationships between the numbers of individuals in samples cannot be determined. The relative abundances for two samples are shown on the left. On the right, there are three scenarios (i.e., scenarios 1 to 3) of different absolute abundances that could yield the relative abundance data that are shown on the left.

One important consideration for any model evaluating the microbiome as a trait is identifying what properties of the microbiome contain information about the host and what properties represent a reflection of the environment or are stochastic. It is unclear how to best quantify microbiomes at the host-species level as a single value. How do we accurately summarize communities as complex as the microbiome? Comparative analyses require large sample sizes and well-estimated trees. Practically speaking, the most cost-effective way to generate microbiome data from hundreds of individuals is by 16S rRNA surveys. However, there are known weaknesses of 16S data that may make it undesirable for the purpose of phylogenetic comparative methods. Consider again functional redundancy or a competitive lottery model of community assembly—the taxa in a sample may not reflect the selection that is determining whether a microbe has a role in a microbiome. Function may be more important and more stable than taxon in microbiomes (3, 14). Using current 16S rRNA methods (without also measuring absolute abundance), means data are strictly compositional, so how taxonomic groups compare in absolute terms across individuals cannot be determined (Fig. 2).

### In my laboratory.

We are investigating how phylogenetic comparative methods perform using 16S data and assessing model fit across neutral models, models that incorporate selection, and models that contain no phylogenetic signal. We are coding the microbiome in terms of composition and diversity, under the null hypothesis that the microbiome contains phylogenetic signal. We are gathering data on the functional capabilities of microbiomes and will code them for phylogenetic comparative method analysis to compare to the models that fit the taxonomic (16S) data. Importantly, our analyses benefit greatly from the wealth of ornithological research on bird life histories, physiology, ecology, and behaviors. Avian microbiome science can also leverage the extensive avian genomics literature to both construct and test hypotheses about the relationships between birds and microbes. One exciting example of this is the OpenWings Project (http://www.openwings.org/)—an ongoing collaboration among 12 major ornithological collections that will gather genomic data (ultraconserved elements [UCEs]) from each of the 10,560 named bird species.

### In the near future.

Microbiome research is interdisciplinary and dependent on the foundational papers and cutting edge research in many fields. As our methods and findings become more sophisticated, I hope to see further integration between theory, phylogenetic methods and models, and community ecology in the context of host-associated microbiomes. We should utilize the wealth of information that has been published in recent decades in the fields of ecology and evolution, but there is also need for development of new models that incorporate microorganism-specific parameters, e.g., horizontal gene transfer. As microbiome research incorporates new markers in addition to 16S and as we find less expensive ways to mine the most meaningful data from microbiomes, it will become clearer how to code the microbiome for comparative analyses.

## INTEGRATION OF THE MICROBE’S PERSPECTIVE WITH THE HOST’S PERSPECTIVE

Microbiomes are hierarchical, and the various layers of interaction may contain conflicting signals. Variation is a major outstanding issue for me, and we are currently collecting data to describe the taxonomic and functional diversity across the entire species range of an endangered saltmarsh specialist, the Saltmarsh Sparrow. Working with the Saltmarsh Habitat and Avian Research Program (SHARP), we have collected data from over 35 populations across the entire breeding range of the species. We will be able to estimate gamma diversity for the species and elucidate the geographic structure of the microbiome. This is also an important basic biodiversity project, as the Saltmarsh Sparrow is projected to be extinct by 2050 and describing their microbiome now, before substantive human intervention, will give us a baseline of their natural microbiome. Gamma diversity is unknown for many, if not most, hosts but could be important for understanding both the community ecology perspective as well as the host trait perspective. How much variation exists in the microbiome? This question could be especially important because as species interactions change in a changing environment, the role of functional redundancy can change as well (15).
